# Energetic and biochemical valorization of cork boiling wastewater by anaerobic digestion

**DOI:** 10.1186/1754-6834-7-67

**Published:** 2014-04-24

**Authors:** Isabel Paula Marques, Luís Gil, Francesco La Cara

**Affiliations:** 1 Laboratório Nacional de Energia e Geologia, I.P., Estrada do Paço do Lumiar 22, Lisboa 1649-038, Portugal; 2 Istituto di Bioscienze e Biorisorse, CNR, Via Pietro Castellino 111, Napoli 80131, Italia

**Keywords:** Anaerobic digestion, Cork boiling wastewater, Methane, Valuable biomolecules, Phenols, Enzymes

## Abstract

**Background:**

In addition to energy benefits, anaerobic digestion offers other interesting advantages. The cork industry is of great environmental, economic and social significance in the western Mediterranean region, with Portugal being the world-leading producer and exporter. Cork boiling wastewater (CBW) is a toxic and recalcitrant organic effluent produced by this sector, which constitutes a serious environmental hazard. However, there is no documented research on anaerobic treatment/valorization performed with this effluent. The work presented here was developed with the aim to use the anaerobic digestion process to convert the CBW polluting organic load into an energy carrier gas and valuable molecules for industry.

**Results:**

No lag phases were observed and a methane yield of 0.126 to 0.142 m^3^ kg^-1^ chemical oxygen demand (COD)_added_ was registered in the mesophilic consortium experiments carried out in batch flasks at 37 ± 1°C. Anaerobic digestion can be advantageously connected to ultrafiltration or electrochemical processes, due to the following: 1) reduction of ellagic acid content and consequent decrease of CBW viscosity; and 2) increase in conductivity after the anaerobic process, avoiding the electrolyte application of the electrochemical process. The improvement of several CBW biochemical features shows that anaerobic digestion may provide additionally useful molecules. The rise in concentration of some of these compounds, belonging to the benzoic acid family (gallic, protocatechuic, vanillic and syringic acids), is responsible for the increase of antiradical activity of the phenolic fraction. Additionally, some enzymatic activity was also observed and while the laccase activity increased in the digested effluent by anaerobiosis, xylanase was formed in the process.

**Conclusions:**

The multidisciplinary approach adopted allowed the valorization of CBW in terms of energy and valuable biomolecules. By exploiting the anaerobic digestion process potential, a novel methodology to toxic and recalcitrant cork processing wastewater was developed.

## Background

Portugal is the world-leading producer and exporter of cork and cork products. Cork is the outer bark of the cork tree (*Quercus suber* L.) and is of high environmental, economic and social importance in the western Mediterranean region where it is produced. Overall data shows the existence of approximately 2.1 million ha of cork oak forests [1]. Portugal has about 34% of this total area (716,000 ha) and produces around 50% (100,000 tons year^-1^) of the overall production. Portugal’s share in total cork exports is significant, at approximately 61% (750 million euro per year, on average, in recent years).

After being harvested from the tree, the cork does not have the necessary elasticity to be processed. It also carries fungi, insects and dirt from the forest and therefore needs pre-treatment. Cork stoppers are the main product and one of the key processing steps is the boiling of cork in water. The pre-treatment operation deals with cleaning/disinfection of the material and leads to an increase of volume and improves workability [2,3]. Boiling is usually carried out for at least 1 hour at a temperature near 100°C. A second boiling operation is sometimes required after stabilization of the boiled cork with a shorter duration. This cork processing step gives rise to high volumes (140 to 1,200 L ton^-1^ cork) [2] of an organic effluent, named cork boiling wastewater (CBW). The composition of CBW depends on the type of cork material to be boiled and the number of boiling cycles, which differs from company to company. This residue has no useful purpose and its polluting potential makes it a serious environmental hazard due to the toxic and recalcitrant organic content.

CBW is characterized by an acid pH and the presence of phenolic compounds and tannins. This presents high acute toxicity for several tested organisms [4,5], which means that CBW has to be treated before disposal. The studies undertaken regarding CBW treatment comprise simple and combined methodologies that have been largely based on physical and chemical processes, such as: ultrafiltration and/or nanofiltration [6-11]; flocculation/flotation/ultrafiltration [12]; ozone and membrane filtration [13]; ozonation combined with hydrogen peroxide and UV radiation [14,15]; solarphoto-Fenton and solarphotocatalysis [16,17]; and Fenton oxidation-coagulation/flocculation [18]. However, many of these treatments are expensive and may give rise to pollutants.

Various techniques have been reported to be developed by applying biological processes, such as the following examples: aerobic bacteria isolation to degrade cork phenolic compounds [19]; chemical oxidation combined with biodegradation (by culture enrichments), which improves the bioavailability of the CBW organic matter [20]; fungal strains used to detoxify the CBW [21]; and ozonation coupled to the aerobic degradation in an activated sludge system [22]. Biological methodologies can effectively contribute to the economic viability of the treatment process and have been successfully applied in the valorization of other agro-industrial effluents similar to CBW. As an example, olive mill wastewater (OMW) is known for its environmental impact and treatment/valorization difficulties [23-25]. Anaerobic digestion is a promising technology for the treatment of the organic effluents and for the simultaneous recovery of its energetic potential through methane production [26,27]. Despite the advantages of the anaerobic process, there is no information in the literature of its application to cork effluents. As with other similar agro-forestry waste materials, there is evidence that CBW contains by-products of high added value that could be useful for industrial applications. In addition to the energetic valorization through anaerobic techniques, particular CBW components may show biological activities of industrial interest. Identification and characterization of these compounds will lead to effluent valorization, contributing to an increase in profit in the cork sector.

**Figure 1:**
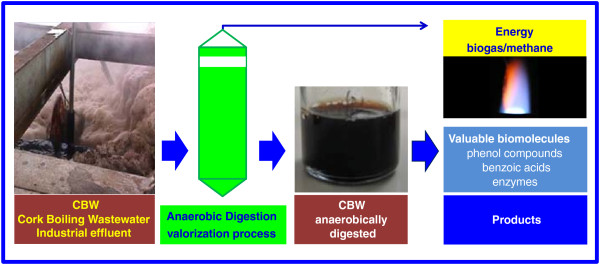
Schematic configuration of the CBW anaerobic digestion process.

The main purpose of this work was to evaluate the possibility of using the anaerobic digestion process to remove the polluting organic load contained in the CBW. Moreover, it was also intended to understand whether this process could be used as a valorization method for this type of effluent, producing an energy carrier gas and different valuable molecules for industry (Figure 1). To be able to evaluate this, the composition of phenolic compounds and enzymes was determined.

## Results and discussion

### Cork boiling wastewater (CBW) as the substrate

CBW is an aqueous and complex effluent of the cork industry that presents an acid pH (5.8) and organic potential of 6.5 kg COD m^-3^, mainly in soluble form (soluble COD = 6.2 kg m^-3^). This wastewater is rich in phenolic compounds (about 1 kg m^-3^) and is deprived of nitrogen content (0.04 kg m^-3^), as reported in Table 1. Similar parameter values have been reported in the literature (see Table 2). This shows that CBW cannot be disposed of without being subjected to prior treatment. This effluent is characterized by an unbalanced composition, low biodegradability, deep dark colour and toxic/inhibiting capacity, which makes it difficult to handle as a substrate. The discrepancy between concentrations of biological oxygen demand (BOD) and COD data leads to low values of BOD/COD ratios, which means that as a substrate CBW is difficult to biodegrade. Table 2 presents various parameters that characterize CBW of different origins. Santos *et al*. [28] obtained BOD/COD ratios varying from 0.2 to 0.3 and Benítez *et al*. [22] reported a value of 0.6.

**Table 1:** Characterization of CBW

**Parameter**	**CBW**^**a**^
pH	5.8 ± 0.0
CODt (kg m^-3^)	6.5 ± 0.1
CODs (kg m^-3^)	6.2 ± 0.1
BOD (kg m^-3^)	-
TS (kg m^-3^)	5.13 ± 0.08
VS (kg m^-3^)	4.05 ± 0.04
TSS (kg m^-3^)	0.58 ± 0.11
VSS (kg m^-3^)	0.15 ± 0.07
Conductivity (mS cm^-1^)	1.5 ± 0.1
Total phenols (kg m^-3^)	1.20 ± 0.00
Total nitrogen (kg m^-3^)	0.04 ± 0.00

^a^Values expressed as an average ± standard deviation of three replicates. BOD, biological oxygen demand; CBW, cork boiling wastewater; CODs, soluble chemical oxygen demand; CODt, total chemical oxygen demand; TS, total solids; TSS, total suspended solids; VS, volatile solids; VSS, volatile suspended solids.

**Table 2:** Characterization of CBW reported in the literature

**Parameter**	**Acero *et al *.**[15]	**Benítez *et al*.**[22]	**Bernardo *et al*.**[6]	**Benítez *et al*.**[7]	**Dias-Machado *et al*.**[20]	**Vilar * et al *.**[17]	**Mendonça *et al*.**[21]	**Santos *et al*.**[28]
pH	4.8	5.4	4.7 to 5.1	4.7	4.7 to 5.7	7.5	4.8 to 5.1	4.6 to 6.2
COD (kg m^-3^)	1.6	1.9	2.29 to 2.60	4.29	2.3 to 4.6	4.7	7.4	2.26 to 11.50
BOD_5_ (kg m^-3^)	0.75	1.15	0.875 to 0.90	1.75	0.49 to 0.74	0.75	1.3	0.5 to 3.5
TOC (kg m^-3^)	-	-	0.671 to 1.57	-	1.2 to 2.0	-	-	1.2 to 2.0
TS (kg m^-3^)	-	-	-	-	-	-	-	1.1 to 1.5
VS (kg m^-3^)	-	-		-	-	-	-	0.85 to 1.0
TSS (kg m^-3^)	0.12	-	-	0.28	-	0.124	-	-
VSS (kg m^-3^)	-	-	-	-	-	0.102	-	-
Conductivity (mS cm^-1^)	-	-	0.934 to 0.935	-	-	2.9	-	1.40 to 1.64
Total phenols (kg m^-3^)	0.305^b^	0.29^b^	0.36 to 0.41^c^	-	0.66 to 0.78^e^	0.74^f^	1.3^g^	(^a^)
Tannins (kg m^-3^)	-	-	0.25 to 0.27^d^	0.761	-	-	-	-
Total nitrogen (kg m^-3^)	-	-	-	-	-	0.31	-	0.06 to 0.2
Toxicity (UT)	-	-	-	-	-	-	3.4 to 12.3	-

^a^Phenolic content as C_6_H_5_OH (0.2 to 0.6 kg m^-3^), phenolic content as caffeic acid (0.48 to 0.58 kg m^-3^), polyphenolic content as gallic acid (1.0 to 3.5 kg m^-3^) and tannins as tannic acid (0.85 to 1.70 kg m^-3^); ^b^total phenols as caffeic acid; ^c^total phenols as tannic acid; ^d^tannins as tannic acid; ^e^polyphenol content; ^f^polyphenols as caffeic acid; ^g^lignin and tannin. BOD_5_, biological oxygen demand of 5 days; CBW, cork boiling wastewater; COD, chemical oxygen demand; TOC, total organic carbon; TS, total solids; TSS, total suspended solids; VS, volatile solids VSS, volatile suspended solids.

The low biodegradability degree is decreased by increasing the load of cork planks boiled and the concentration of tannins in the wastewater [28]. On the other hand, acute and chronic toxicity tests performed with organisms of different trophic levels classify this wastewater as ecotoxic [5,29]. The CBW inhibiting capacity is mainly related to the acid pH, lack of nitrogen and the presence of phenolic compounds (19% of the total COD, as presented in Table 1).

### Gas production

The methane production obtained in the biodegradability tests performed at 3 and 6 kg COD m^-3^ of CBW was measured over 44 days. No lag phases were observed in the presence of CBW. In the batch experiments performed with 3 kg COD m^-3^, the maximum methane production (15 mg COD-CH_4_ batch^-1^) was attained after 15 days and was kept stable until the end of the experimental time. In the batch experiment performed with 6 kg COD m^-3^, most of the methane was produced in the first 15 days of the experimental run, having evolved to 27 mg COD-CH_4_ batch^-1^ at the end.The present data suggest that CBW can be regarded as an interesting substrate for the production of biogas through a biotransformation process.

More diluted wastewater (3 kg COD m^-3^) shows higher conversion efficiency than the original substrate (6 kg COD m^-3^): 0.142 m^3^ CH_4_ kg^-1^ COD_added_ ± 0.014 versus 0.126 m^3^ CH_4_ kg^-1^ COD_added_ ± 0.013. Comparison of these data with the average value of 0.131 m^3^ CH_4_ kg^-1^ COD of influent, obtained in the anaerobic hybrid reactor digesting the raw OMW in a continuous mode [24], shows that they are of the same order of magnitude. Although there was a lower methane yield, the highest concentration of CBW tested did not inhibit or negatively influence the gas production. Using a double CBW concentration, the volume of methane produced was also double the volume obtained with 3 kg COD m^-3^.

Biogas/methane is an energy source and the cork processing activity is an important energy consumer. Thus, the gas flow obtained from the anaerobic digestion treatment can be applied in the cork processing operation. The estimated total volume of CBW produced yearly in Portugal is 30,374 m^3^ and the annual energy potential of this wastewater could amount to a global energy production in a combined heat and power (CHP) system of greater than 165,000 kWh. The digested material is detoxified and can be used advantageously in agriculture. The remaining organic matter and nutrients are a useful additive for the cork oak forests.

### CBW organic load removal

The CBW methane yield obtained at 3 and 6 kg COD m^-3^ (0.142 and 0.126 m^3^ CH_4_ kg^-1^ COD_added_) [30] corresponds to a COD removal of 40% and 36%, respectively. These results are comparable to others found in the literature also using biological methods. Benítez *et al*. [22] used an activated sludge process and obtained a COD removal of 13% to 37%; and Mendonça *et al*. [21] ran fungi degradation tests, which obtained removals of 48% to 62%, and sequential biodegradation experiments, which resulted in a slight increase in COD removal that did not exceed about 10%. In the present case, an organic fraction of approximately 60% remained to be degraded under tested anaerobic conditions. This might be related to the presence of none or difficult biodegradable substances, or to inhibition of the microbial consortium [31-33]. The first case is more likely to have occurred in the present research, where the effect of the presence of recalcitrant compounds was more important than the development of any inhibiting process. This consideration is supported by the fact that no lag phases were detected in the presence of this potentially toxic/inhibitor substrate. The utilization of an adapted biomass to the OMW treatment may have contributed positively to the good performance of digestion start-up units operating with CBW. The anaerobic digestion could provide a detoxified stream with a lower loading rate than the initial CBW, once proven that it is possible to digest this difficult substrate. Consequently, the anaerobic process makes the effluent environmentally friendlier and allows a more appropriate use of the large volumes of wastewaters produced every year.

### Volatile fatty acids (VFA) and pH

The good operational behaviour of the units is also confirmed by the concentration and composition of volatile fatty acids (VFA) and pH values (Table 3). The microbial population is sensitive to the presence of inhibitory substances and also to pH [31]. Neutral/basic pH values are favourable to the development of anaerobic digestion, while acid pH conditions give rise to problems in managing the process. No acid pH values were recorded at the end of the experiments and a slight pH increase was observed during the process. With regard to the VFA, the results demonstrated that acetic acid was the only short-chain carboxylic acid present in the original CBW, inoculum and effluents of both operating conditions. Most of the methane produced had its origin in acetic acid and this gives an indication of the stability of the process. It is noted that the VFA concentrations in the original CBW and in the inoculum (AD_0_) are the highest and are similar. The initial VFA concentrations did not cause acidification of the medium and removal of acids occurred during the process. A decrease of 36% of VFA (acetic acid) concentration was observed with 6 kg COD m^-3^. It was possible to digest CBW by a biological process such as anaerobiosis. However, it must be emphasized that the anaerobic experiments worked without any pre-treatment of the reactor influent and only pH correction was undertaken. Considering the experience obtained in successfully handling the anaerobic digestion of unbalanced and inhibiting industrial effluents, such as the olive oil production effluent (OMW), without any pre-treatment or correction of the substrate, the authors are continuing research in order to also avoid the pH correction step on CBW, and to attain a simpler and cheaper conversion process.

**Table 3:** Experimental parameters

**Parameter**	**CBW**	**AD0**	**AD3**	**AD6**
pH	7.20 ± 0.10	8.23 ± 0.09	7.67 ± 0.02	7.52 ± 0.06
VFA (kg m^-3^)	0.215 ± 0.023	0.185 ± 0.036	0.178 ± 0.048	0.137 ± 0.013
Total phenols (mg mL^-1^)	1.20 ± 0.02	0.05 ± 0.00	0.90 ± 0.00	1.41 ± 0.02
OD (254 nm)	43.50 ± 0.22	8.41 ± 0.64	29.08 ± 0.66	43.96 ± 0.00
DPPH inhibition (%)	74.00 ± 0.84	78.90 ± 0.42	76.07 ± 1.56	80.80 ± 1.06
EC^a^_50_ (antiradical activity)	7.20 ± 0.11	2.22 ± 0.08	5.90 ± 0.08	8.74 ± 0.06
Conductivity (mS cm^-1^)	1.50 ± 0.10	4.00 ± 0.11	4.60 ± 0.00	5.40 ± 0.11

^a^Antiradical activity defined as the amount of antioxidant (expressed as μg of total polyphenols) necessary to decrease the initial DPPH concentration by 50%. Values presented as an average ± standard deviation. AD0, blank anaerobic digestion assay; AD3, anaerobic digestion assay at 3 kg COD m^-3^ of CBW; AD6, anaerobic digestion assay at 6 kg COD m^-3^ of CBW; CBW, cork boiling wastewater, reactor influent; DPPH, 2,2-diphenyl-1-picrylhydrazyl; EC_50_, half maximal effective concentration; OD, optical density; VFA, volatile fatty acid.

### Phenolic compounds: colour and UV absorption

Data on total phenolic compounds reveal that their concentration in the original fluid was not altered by the anaerobic process under the operational conditions used in the tests (see Table 3). The values reported indicate an increase of approximately 18% in the amount of phenols at the end of the experimental run (AD6). Comparing results obtained in the experiments carried out with CBW, the unit with a lower concentration (3 kg COD m^-3^) presents a total concentration of phenols that corresponds to 64% of the total phenol concentration found in the 6 kg COD m^-3^ unit.

Referring to colour, Field and Lettinga [34] studied the effect of oxidative colour on methanogenic toxicity and biodegradability of a synthetic phenolic solution. They found that coloured compounds were not biodegradable and their presence did not affect the biodegradability of colourless compounds. In accordance with the above, as the substrate was visually dark at the end of the experiment, this suggests that the phenolic compounds were still present. Moreover, the phenolic fraction holding onto the CBW may not have contributed to the methane production.

Concerning absorbance, no considerable change on the substrate was observed after anaerobic digestion under test conditions. Identical values were obtained (43.5 versus 43.96 optical density (OD), Table 3). The absorbance at 254 nm was used to interpret the changes in the aromatic content of the treated wastewater because 254 nm corresponds to the wavelength at which the aromatic and unsaturated compounds show the maximum absorption. The results obtained indicate a minor change in CBW UV absorbance, suggesting that the amount of aromatic content did not change by anaerobiosis. The OD at 3 kg COD m^-3^ corresponds to 66% of the values obtained at 6 kg COD m^-3^ (see Table 3). These results are in accordance with the data on phenolic compounds as they are the main contributors to the UV absorbance at 254 nm.The increase of the concentration of total phenols without change of the UV absorbance value of the digested flow may be explained by the maintenance of the pH values into the alkaline range during the process. As a result, the solubility of the phenolic compounds is maintained and the CBW UV absorbance is not subject to major change.

According to Dias-Machado *et al*. [20], CBW polyphenols are not readily biodegradable. The biodegradation rate was not improved even by using enrichment cultures able to degrade tannic acid and other polyphenols in synthetic culture medium. To improve the rate value, the technique of inorganic nutrient supplementation was used, and nitrogen and phosphorus and an inoculum density 100 times higher was used. Considering the possibility of the polyphenol unavailability for biodegradation and their inhibitory effects on bacteria, Dias-Machado *et al*. [20] suggested a pre-treatment (chemical oxidation) of CBW to promote the biodegradation.

In our study, the results obtained show that a biological process can be used as a first step of CBW treatment by using the anaerobic digestion process. One of the advantages of this process is the recovery of the methane production from the degradation of the CBW organic fraction. It was observed that CBW contains a concentration of phenolic compounds higher than 1 kg m^3^, UV absorbance of the substrate did not alter significantly and the dark colour persisted. This means that a significant fraction of organic aromatic matter may remain without being degraded by anaerobiosis. Consequently, further treatment might be needed and anaerobic digestion could be connected to the electrochemical oxidation as a post-treatment stage. The feasibility of a two-step process for the treatment and valorization of olive oil effluent was proven in recent work. Results show that the phenolic fraction, the dark colour and the remaining COD of the anaerobically digested effluent can be removed through the electrochemical process [35,36]. Furthermore, the obtained flow devoid of organic matter could be discharged or used for other applications.

### Antiradical potential

An antiradical activity value of half maximal effective concentration (EC_50_) = 7.2 μg was measured in CBW (see Table 3). Similarly to total phenolic compounds, an increase in the antiradical activity was observed after anaerobic digestion, suggesting that the phenol composition varied over the process. Formation of phenols with a different chemical structure may have occurred during the process development. These types of molecules are valuable and useful for several industrial applications. The production of phenolic compounds with a higher antiradical activity as well as the extraction of natural antioxidants by anaerobiosis is a very interesting feature for the industrial valorization of CBW.

### CBW conductivity

A relevant point is the high conductivity of the original CBW that may be attributed to the concentration of potassium salts contained in such effluents [17]. A potassium concentration of 1.6 g L^-1^ and conductivity of 2.9 mS cm^-1^ have been reported [17]. Values in the range of 1.4 to 3.2 mS cm^-1^ of the original CBW have also been indicated by other authors [18,37]. In the present work, the substrate had a conductivity of 1.5 mS cm^-1^ (approximately 0.01 M as KCl).

The conductivity of the digested effluent in both operational conditions (3 and 6 kg COD m^-3^, Table 3) was 4.6 and 5.4 mS cm^-1^, respectively. These values are considered high, which might be related to the biotransformation process as well as the inoculum that contains higher ion concentration than the original CBW. The presence of high conductivity values is of great interest in the case of the two-step process application, using the electrochemical oxidation as a post-treatment of anaerobic digestion. The required addition of electrolyte for the electrochemical process is decreased, or even avoided. This leads to a cheaper electrochemical phase and promotes the obtained recycle flow to the cork boiling process.

### Composition of phenolic compounds

Ten phenolic compounds (gallic acid, protocatechuic acid, caffeic acid, vanillic acid, syringic acid, ellagic acid, *para*-coumaric acid, ferulic acid, *ortho*-coumaric acid and *trans*-cinnamic acid) were identified by HPLC analysis in the cork processing wastewaters, inoculum (blank) and two different digested effluents. The quantitative evaluation of these compounds is reported in Table 4. The major component of the phenolic fraction of CBW is ellagic acid, at a concentration of 96.5 μg mL^-1^, followed by gallic acid present in a much lower amount (19.5 μg mL^-1^). The results obtained for the most representative phenolic compounds of CBW are quite different, but comparable to those reported in the literature [12,37]. Concerning the gallic, protocatechuic, vanillic and syringic acids, which belong to the benzoic acid family, it was observed that their amounts in treated samples differ from the original CBW and an increase was registered in the 6 kg COD m^-3^ assay.

**Table 4:** HPLC analysis of phenolic compounds in CBW experiments

**Phenolic compound (μg mL**^**-1**^**)**	**CBW**	**AD0**	**AD3**	**AD6**
Gallic acid	19.50 ± 3.71	4.17 ± 0.26	15.47 ± 4.08	22.43 ± 3.97
Protocatechuic acid	8.50 ± 1.02	0.64 ± 0.09	5.07 ± 1.59	9.82 ± 1.33
Caffeic acid	2.14 ± 0.15	0.59 ± 0.44	1.15 ± 0.21	1.77 ± 0.36
Vanillic acid	2.00 ± 0.19	tr	1.15 ± 0.37	1.91 ± 0.36
Syringic acid	tr	nd	tr	1.10 ± 0.99
Ellagic acid	96.50 ± 11.50	2.49 ± 0.39	22.3 ± 3.48	41.80 ± 8.49
*p*-Coumaric acid	4.10 ± 0.26	1.10 ± 0.18	1.77 ± 0.28	2.47 ± 0.40
Ferulic acid	6.50 ± 0.78	1.02 ± 0.08	1.55 ± 0.37	5.60 ± 0.98
*o*-Coumaric acid	3.80 ± 0.29	tr	1.17 ± 0.32	4.51 ± 0.33
*trans*-Cinnamic acid	3.70 ± 0.37	2.16 ± 0.26	2.10 ± 0.29	3.13 ± 0.63

Values presented as an average ± standard deviation. AD0, blank anaerobic digestion assay; AD3, anaerobic digestion assay at 3 kg COD m^-3^ of CBW; AD6, anaerobic digestion assay at 6 kg COD m^-3^ of CBW; CBW, cork boiling wastewater, reactor influent; HPLC, high performance liquid chromatography; nd, not detected; tr, traces.

On the contrary, the phenolic compounds belonging to the hydroxycinnamic acid family (caffeic, *para*-coumaric, ferulic, *ortho*-coumaric and *trans*-cinnamic acid) showed in both assays (3 and 6 kg COD m^-3^) a significant decrease compared with those of CBW. However, there is an exception in the case of the *ortho*-coumaric acid concentration, which increased 19% in the 6 kg COD m^-3^ assay.

A particular observation must be made for the ellagic acid, which presents a concentration decrease of 57% (6 kg COD m^-3^ assay). Ellagic acid is distinguished from the other ten identified phenolic compounds in CBW due to hydrophobicity and higher molecular weight (MW). Ellagic acid has a MW of 302.2 Da, which is high compared to less than 200 Da for the other CBW phenolic compounds. The capability of the anaerobic digestion process for removing large quantities of this compound is of great importance. Ellagic acid was described as the main soluble molecule contained in the CBW responsible for membrane fouling due to its strong hydrophobic interaction [37]. Thus, the observed reduction of the ellagic acid content promotes a viscosity decrease of the CBW flow and allows the application of technologies such as ultrafiltration.

The additional production of valuable and useful molecules for several industrial applications is also possible after anaerobic digestion, which is another relevant aspect to consider. A similar increase in the total phenolic compounds concentration and antiradical activity was verified, indicating that the compounds with a higher industrial interest were formed. During the process, the concentration of the benzoic acid family (gallic, protocatechuic, vanillic and syringic acids) and *ortho*-coumaric acid improved. Phenolic compounds are significant components for the human diet due to their potential antioxidant activity, their ability to diminish oxidative stress-induced tissue damage resulting from chronic diseases and their potential utilization in cancer therapy [38].

### Enzymatic activities

Table 5 reports enzymatic activities of lipase, glucosidase, laccase, cellulase and xylanase in the original CBW, inoculum and digesters of the two operational conditions (AD3 and AD6). Lipases catalyze the hydrolysis of ester linkages in lipids and are therefore employed in food technology (mainly in the dairy industry) and also in the detergent, pharmaceutical, cosmetic and leather industries. Laccases exhibit low substrate specificity and good stability in the presence of various potentially denaturing agents, thus suggesting their possible use in several applications. Glucosidases, cellulases and xylanases are enzymes that hydrolyze carbohydrates, and are utilized in the pulp and paper and food industries. One of the main applications of these enzymes is in the conversion of carbohydrates in biomass to fermentable sugars, and their use is essential for developing commercially competitive biological processes for making cellulosic ethanol.

**Table 5:** Enzymatic activities in CBW experiments

**Enzymes (U mL**^**-1**^**)**	**CBW**	**AD0**	**AD3**	**AD6**
Lipase	3.63 ± 0.04	0.83 ± 0.01	0.66 ± 0.02	0.84 ± 0.01
β-Glucosidase	tr	0.16 ± 0.00	nd	tr
Laccase	0.79 ± 0.02	0.99 ± 0.02	1.97 ± 0.02	2.10 ± 0.01
Cellulase	0.21 ± 0.01	tr	0.08 ± 0.00	0.12 ± 0.00
Xylanase	tr	nd	0.10 ± 0.00	0.15 ± 0.01

Values presented as an average ± standard deviation. AD0, blank anaerobic digestion assay; AD3, anaerobic digestion assay at 3 kg COD m^-3^ of CBW; AD6, anaerobic digestion assay at 6 kg COD m^-3^ of CBW; CBW, cork boiling wastewater, reactor influent; nd, not detected; tr, traces.

Cork processing wastewater is an effluent containing several enzymatic activities, with lipase activity being the most representative (3.63 U mL^-1^). Laccase and cellulase activities are also detected but with less occurrence. Concerning the process, a decrease of lipase activity is observed revealing that the greatest lipolytic capacity of CBW is lost during the anaerobic digestion. Identical values have been registered at the end of AD0 and AD6, while approximately 80% of this amount was recorded in AD3. β-Glucosidase activity has not been produced in all tests except in the case of the inoculum batch, presenting a value of 0.16 U mL^-1^. An interesting result is obtained for the laccase activity, which increased in both treated effluents. An increment of about 2.5-fold was registered at AD6. Regarding the complex carbohydrate hydrolyzing enzymes (cellulase and xylanase), using chromogenic substrates, the results of quantitative analyses showed an activity decreasing for cellulase and increasing for xylanase in both treated effluents as compared to the original CBW.

The production of enzymes with commercial interest offers an additional interesting opportunity for the biotechnological valorization of the effluent under study. The enzymes obtained by the wastewater treatments include lipases, laccases, carbohydrate hydrolyzing enzymes (glucosidases, cellulases and xylanases), and so on [39]. Enzyme presence was already reported in processes using OMW as the substrate [40]. Comparatively, the original CBW contains fewer enzymes compared to OMW. Considering the present data (Table 5), it is possible to infer that CBW is characterized by the presence of lipase activity. In addition, it was observed that while the concentration of laccase activity was increased in the digested effluent by anaerobiosis, xylanase was formed during the process. The enzyme activities recorded during the anaerobic digestion are of great industrial interest as the enzymes may be utilized in several commercial applications, for example paper manufacturing, wine stabilization and wastewater treatment.

## Conclusions

The effluent from cork processing can be treated and valorized by a biological process such as anaerobic digestion. The energetic potential of CBW has direct application in cork processing operations, while the digested flow can be used in cork oak forests to increase the organic matter of the soil. In this work, a novel treatment and valorization approach to the toxic and recalcitrant CBW was developed.

Anaerobic digestion is mainly known as a technology dedicated to wastewater/residue treatment with an energy benefit. This work shows that in addition to energy, anaerobiosis should be regarded as a means of providing other interesting profits, due to its capacity in removing/decreasing or increasing/forming various compounds.

In terms of CBW treatment, anaerobic digestion can be advantageously associated with other technological processes. The observed reduction of the ellagic acid content in CBW by anaerobiosis promotes a viscosity decrease of the flow and allows the application of technologies such as ultrafiltration. Moreover, the conductivity increase provides cheaper conditions to completely remove the remaining organic load, using electrochemical techniques as a post-treatment of anaerobic digestion. It also promotes the recycle flow to the cork industrial unit.

The energetic and agricultural values of CBW through anaerobic digestion result from the obtained methane and the increase of nutrient concentration in the digested substrate, respectively. The additional production of valuable and useful molecules for several industrial applications is another interesting aspect offered by anaerobic digestion. Identical increases in the total phenolic compound concentration and antiradical activity were obtained simultaneously by anaerobic digestion, indicating that compounds with a higher industrial interest were formed. During the process, the concentration of the benzoic acid family (gallic, protocatechuic, vanillic and syringic acids) and *ortho*-coumaric acid improved. An increase of enzyme activities was also registered and while laccase activity increased in the digested effluent by anaerobiosis, xylanase was formed in the process. These enzymes may find use in several commercial applications.

## Methods

### Experimental set-up and procedure

The biodegradability experiments were performed in closed vials with a total volume of 35 cm^3^ and a working volume of 12.5 cm^3^. The inoculum was added to the vials at a final concentration of approximately 5 kg volatile suspended solids (VSS) m^-3^. The basal medium was made up with demineralized water and sodium bicarbonate (3 kg m^-3^). The batch tests were performed, using the diluted CBW (50%) and the original that resulted into substrate concentrations of 3 and 6 kg COD m^-3^ (anaerobic digestion assay: AD3 and AD6, respectively). No substrate was added to the blank control units (anaerobic digestion assay: AD0). The batch vials were flushed with N_2_, closed with a butyl rubber stopper and sealed after transferring the inoculum, the basal medium and the substrate. The tests were performed at 37°C, in triplicate. A handheld pressure transducer was used to measure the gas production throughout the experimental time. A volume of approximately 30 μL of biogas was released by each pressure measurement, which represented an insignificant fraction of the total biogas produced in the experiment. The applied technique was based on the method developed by the Microbiology Department of the National University of Ireland (Galway, Ireland), under the supervision of Professor Emer Colleran [41,42].

The methane accumulated in the vessel headspace was measured by gas chromatography, as described in the Analytical methods section, by collecting 500 μL of sample volume using a gas-tight syringe. Methane production was corrected for standard temperature and pressure (STP) conditions. The amount of methane produced was converted to its COD equivalent (mg COD-CH_4_) considering the biochemical methane potential (350 L CH_4_ kg^-1^ COD). The methane yield was expressed as the ratio between the methane produced and the COD added to the batch vials (m^3^ CH_4_ kg^-1^ COD_added_).

### Inoculum and substrate

The sludge obtained from a hybrid reactor treating OMW, as described elsewhere, was used as the inoculum [23,24]. It was pre-incubated at 37°C in order to deplete the residual biodegradable organic material. The cork boiling effluent was obtained from a cork industry facility in Alcácer do Sal, Portugal. The substrate was stored at 4°C until use. CBW was characterized and the pH adjusted to neutral values (7.0 to 7.2) with NaOH before using. The CBW characteristics are summarized in Table 1.

### Analytical methods

Total and soluble chemical oxygen demands (CODt and CODs, respectively) and total nitrogen were evaluated using Spectroquant® test kits (Merck, Whitehouse Station, NJ, USA), and total and volatile solids, and total and volatile suspended solids (TS, TV and TSS, VSS, respectively) were measured according to standard methods [43]. Conductivity was evaluated by a conductivity meter (EcoTestr, OAKTON Instruments, Vernon Hills, IL, USA). Total phenolic compound concentrations were expressed as mg of caffeic acid equivalent and were determined via a modified Folin–Ciocalteu method [44].

### Gas chromatography analysis

Gases were separated in a 1/8” × 3 m Porapak® column (80 to 100 mesh) and analyzed with a thermal conductivity detector in a Varian 3800 chromatograph (Varian, Walnut Creek, CA, USA), with column, injector and detector at 50°C, 60°C and 100°C, respectively. VFA (acetate, propionate, butyrate, iso-butyrate, iso-valerate and valerate) were analyzed using a gas chromatograph (Hewlett-Packard 5890, Hewlett-Packard, Palo Alto, CA, USA), equipped with a flame ionization detector and a 2 m × 2 mm Carbopack B-DA/4% Carbowax 20 M (80 to 120 mesh) column. Helium was used as the carrier gas (30 mL min^-1^), and the temperature of the column, injector and detector were 170°C, 175°C and 250°C, respectively.

### UV absorption

The UV-absorbing organic compounds in a sample absorbed UV light in proportion to their concentration [45,46]. UV absorption was measured at 254 nm using a Cary 50 (Varian) spectrophotometer with a 1 cm path length quartz cell. Samples were centrifuged for 15 min at 3,500 rpm and the recovered supernatant was filtered through cellulose nitrate membrane filters (pore size of 0.45 μm) to control variations in UV absorption caused by particles [15]. The samples were diluted with several amounts of distilled water (usually in a ratio 1:50) to a volume that could produce a UV absorbance between 0.05 and 0.95.

### Antiradical activity

The antiradical activity was defined as the amount of antioxidant (expressed as μg of total polyphenols) necessary to decrease the initial 2,2-diphenyl-1-picrylhydrazyl (DPPH) concentration by 50% (EC_50_ = efficient concentration). The antiradical activity of the raw and digested CBW was determined by a modification of the method described by von Gadow *et al*. [47]. Briefly, 1 mL of a 6 × 10^-5^ M methanolic solution of DPPH was added to 10 mL of a methanolic solution of CBW samples mixed and placed in 1 cm glass cuvettes. The decrease in absorbance at 515 nm was determined continuously with data acquisition at 2 s intervals with a spectrophotometer Varian Cary 50 for 16 min (until the absorbance stabilized). The inhibition percentage (IP) of the DPPH radical by the phenolic compounds of the CBW extracts was calculated according to the formula:

$$\mathrm{IP}\;=\;\left[\left(\mathrm{AC}\left(0\right)-\mathrm{AC}\left(t\right)\right)/\mathrm{AC}\left(0\right)\right]\times 100$$

where AC(0) is the absorbance of the control at t = 0 min and AC(t) is the absorbance of the reaction solution at t =16 min [48].

### HPLC analysis of phenolic compounds

Aliquots of 0.1 mL of each sample were diluted in 5 mL of acid methanol (70:29:1 of methanol:water:HCl) and incubated at 37°C for 30 min in a rotary shaker. The suspension was centrifuged for 15 min at 3,500 rpm, and the supernatant was recovered and used for polyphenol assay. HPLC/UV analysis of single phenolic compounds was performed utilizing a 250 × 4.6 mm (5 μm) C18 Hypersil column (Thermo Electron Corporation, Bellefonte, PA, USA), used with a SecurityGuard precolumn (Phenomenex, Macclesfield, UK) and a C18 cartridge in combination with a ThermoFinnigan Surveyor HPLC system (solvent degasser, quaternary pump, thermostatically controlled column oven set at 25°C, a photodiode array detector set to collect overall data from 200 to 600 nm, and selected wavelengths of 230, 254 and 280 nm). The solvent flow rate was 0.9 mL min^-1^ and the mobile phase was a four-step linear solvent gradient system (0 to 30 min, 10% B; 30 to 35 min, 55% B; 35 to 40 min, 100% B; 40 to 45 min, 100% B) using 2% acetic acid in water as solvent A and 0.5% acetic acid in 50% acetonitrile as solvent B. Identification of phenolic compounds in the CBW extracts was performed by HPLC-UV, comparing the relative retention times and UV spectra with those of standard solutions [49].

### Enzyme assays

Lipase activity was estimated using a spectrophotometric assay with *p*-nitrophenyllaurate (*p*NPL) as a substrate, which was dissolved in acetonitrile at a concentration of 10 mM. Subsequently, ethanol and 50 mM potassium phosphate buffer (pH 7.5) were added to a final composition of 1:4:95 (v/v/v) of acetonitrile:ethanol:buffer, respectively [50]. The CBW samples were centrifuged at 12,000 rpm for 5 min and 0.05 mL of the supernatant was added to the substrate solution (0.95 mL). The mixture was then incubated at 37°C. After 5 min, enzyme activity was measured by monitoring the change in absorbance at 405 nm that represents the amount of released *p*-nitrophenol (*p*NP). All measurements were carried out under first-order reaction conditions, that is, with the catalyst in excess of ester. One unit of lipase activity is defined as the amount of enzyme releasing 1 μmol *p*NP per min under the assay conditions (ϵ_405_ = 16,860 M^-1^ cm^-1^). β-Glucosidase activity was estimated with a similar method using a spectrophotometric assay with *p*-nitrophenyl-β-D glucopyranoside (*p*NPG) as a substrate, which was dissolved in distilled water at a concentration of 5 mM. The CBW samples were centrifuged at 12,000 rpm for 5 min, and 0.05 mL of the supernatant was added to a mixture containing 0.5 mL of 100 mM potassium phosphate buffer (pH 7.5), 0.2 mL of substrate solution and 0.25 mL of distilled water. The mixture was incubated at 37°C. After 15 min, enzyme activity was measured by monitoring the change in absorbance at 405 nm that represents the amount of released *p*NP. One unit of β-glucosidase activity is defined as the amount of enzyme releasing 1 μmol *p*NP per min under the assay conditions (ϵ_405_ = 16,860 M^-1^ cm^-1^).

Laccase activity was determined by oxidation of 2,2’-azino-bis(3-ethylbenzthiazoline-6-sulfonic acid) (ABTS) as a substrate. The reaction mixture contained 0.1 mL of 20 mM ABTS in distilled water, 0.1 mL of 0.1 M sodium acetate buffer (pH 5.0), 0.05 mL of CuSO_4_, 20 mM 0.74 mL of distilled water and 0.01 mL of sample supernatant centrifuged at 12,000 rpm for 5 min. The mixture was incubated at 37°C for 5 min. Oxidation of ABTS was followed by an absorbance increase at 420 nm. The enzyme activity was expressed in units defined as the amount of enzyme oxidizing 1 μmol of ABTS min^-1^ (ϵ_420_ = 36,000 M^-1^ cm^-1^) [51]. Concerning the carbohydrate hydrolyzing enzymes (cellulase and xylanase), quantitative enzymatic analyses were performed based on the use of chromogenic substrates that allowed the quantification of the units of the enzymes present in the samples.

Cellulase activity was evaluated at 50°C and pH 7.5 utilizing as substrate the Azo-carboxymethylcellulose (Azo-CMC) dyed with RemazolBrilliant Blue R(RBB) [52]. Next, 150 μL of the samples were incubated with 150 μL of substrate for 10 min at 40°C. After the incubation, 700 μL of ethanol was added and the mixture was maintained at room temperature for 10 min. The samples were centrifuged at 12,000 rpm for 10 min and the enzyme activity was evaluated by monitoring the change in absorbance at 590 nm. The unit of cellulase activity, in the assay conditions, was determined by reference to a standard curve of *Trichoderma* sp. enzyme on Azo-CMC.

Xylanase activity was evaluated at 50°C and pH 7.5 utilizing as substrate the RBB-xylan. Next, 150 μL of the samples were incubated with 150 μL of substrate for 10 min at 40°C. After the incubation, 700 μL of n-propanol was added and the mixture was maintained at room temperature for 5 min. The samples were centrifuged at 12,000 rpm for 10 min and the enzyme activity was evaluated, as in the cellulase assay, by monitoring the change in absorbance at 590 nm. One unit of xylanase activity is defined as the amount of enzyme required to release one μmol of D-xylose reducing sugar equivalents per minute from wheat arabinoxylan in the assay conditions.

## Abbreviations

ABTS: 2,2’-Azino-bis(3-ethylbenzthiazoline-6-sulfonic acid; AD: Anaerobic digestion; BOD: Biological oxygen demand; CBW: Cork boiling wastewater; CHP: Combined heat and power; CMC: Carboxymethyl cellulose; COD: Chemical oxygen demand; DPPH: 2,2-Diphenyl-1-picrylhydrazyl; EC50: Half maximal effective concentration; HPLC: High performance liquid chromatography; IP: Inhibition percentage; MW: Molecular weight; OD: Optical density; OMW: Olive mill wastewater; pNP: *p*-Nitrophenol; pNPG: *p*-Nitrophenyl-β-D glucopyranoside; pNPL: *p*-Nitrophenyl laurate; RBB: Remazol Brilliant Blue R; STP: Standard temperature and pressure; TOC: Total organic carbon; TS: Total solids; TSS: Total suspended solids; VFA: Volatile fatty acid; VS: Volatile solids; VSS: Volatile suspended solids.

## Competing interests

The authors’ declare that they have no competing interests.

## Authors’ contributions

IPM conceived of the study, designed the anaerobic reactor and the experiments, supervised the work, collaborated in the evaluation of the data and drafted the manuscript. LG was involved in the CBW sample collection, wrote part of the manuscript related to the cork sector/industry and assisted in drafting the manuscript. FLC performed the experimental research on biomolecules, participated in the designed experiments, collaborated in the evaluation of the data, wrote part of the manuscript related to the biomolecule evaluation and assisted in drafting the manuscript. All authors critically revised the draft, and read and approved the final manuscript.
